# Synchronously Strengthen and Toughen Polypropylene Using Tartaric Acid-Modified Nano-CaCO_3_

**DOI:** 10.3390/nano11102493

**Published:** 2021-09-24

**Authors:** Junlong Yao, Hanchao Hu, Zhengguang Sun, Yucong Wang, Huabo Huang, Lin Gao, Xueliang Jiang, Xinrui Wang, Chuanxi Xiong

**Affiliations:** 1 Hubei Key Laboratory of Plasma Chemistry and Advanced Materials, Key Laboratory for Green Chemical Process of Ministry of Education, School of Materials Science and Engineering, Wuhan Institute of Technology, No. 206 Guanggu 1st Road, Wuhan 430205, China; yaojunlong01@126.com (J.Y.); withuhanchao@163.com (H.H.); ycwang_wit@163.com (Y.W.); jiangxueliang2008@163.com (X.J.); wangxr2233@126.com (X.W.); 2 Ministry-of-Education Key Laboratory for the Green Preparation and Application of Functional Materials, Hubei University, No. 368 Friendship Avenue, Wuhan 430062, China; zgsun_polymer@126.com; 3 School of Chemistry and Environmental Engineering, Jianghan University, No. 8 Sanjiaohu Road, Wuhan 430056, China; 4 State Key Laboratory of Advanced Technology for Materials Synthesis and Processing, School of Materials Science and Engineering, Wuhan University of Technology, Luoshi Road 122, Wuhan 430070, China

**Keywords:** polypropylene, calcium carbonate, tartaric acid, modification, dispersion

## Abstract

In order to overcome the challenge of synchronously strengthening and toughening polypropylene (PP) with a low-cost and environmental technology, CaCO_3_ (CC) nanoparticles are modified by tartaric acid (TA), a kind of food-grade complexing agent, and used as nanofillers for the first time. The evaluation of mechanical performance showed that, with 20 wt.% TA-modified CC (TAMCC), the impact toughness and tensile strength of TAMCC/PP were 120% and 14% more than those of neat PP, respectively. Even with 50 wt.% TAMCC, the impact toughness and tensile strength of TAMCC/PP were still superior to those of neat PP, which is attributable to the improved compatibility and dispersion of TAMCC in a PP matrix, and the better fluidity of TAMCC/PP nanocomposite. The strengthening and toughening mechanism of TAMCC for PP involves interfacial debonding between nanofillers and PP, and the decreased crystallinity of PP, but without the formation of β-PP. This article presents a new applicable method to modify CC inorganic fillers with a green modifier and promote their dispersion in PP. The obtained PP nanocomposite simultaneously achieved enhanced mechanical strength and impact toughness even with high content of nanofillers, highlighting bright perspective in high-performance, economical, and eco-friendly polymer-inorganic nanocomposites.

## 1. Introduction

Polypropylene (PP), a thermoplastic polymerized by propylene monomer, has been widely used in automobiles, appliances, packaging, and other fields [1,2], because of its good chemical resistance, satisfactory heat resistance, electrical insulation, high mechanical strength and wear resistance, etc. It also has favorable processability and can adapt to conventional plastic processing technology, such as injection, extrusion, blow molding, calendering, rotary molding, etc., and has become one of the most commercialized and popular thermoplastics [3]. However, poor impact resistance has fairly hindered the further application of PP [4].

In recent years, many studies and reports have conducted in-depth research on the basic theory and application of polypropylene toughening. There are principally two toughening methods for PP, blending rubber or elastomer, and adding inorganic rigid particles with PP [5,6]. For the former, the blending of rubber or elastomer with PP can significantly increase the toughness of PP, but at the same time, the modulus, strength, and hot deformation temperature of PP composites would inevitably decrease [5,7]. In comparison, the addition of inorganic rigid particles can not only considerably reduce the cost, but also bring positive effects on the prepared composites, such as good heat resistance, low shrinkage, good dimensional stability, and high stiffness [2,6]. More importantly, the addition of nano-sized particles (namely, nanofillers) can achieve comprehensively enhanced mechanical performance in nanocomposites. Thanks to mature manufacture technology, low price, and abundant reserves, calcium carbonate (CaCO_3_) nanoparticles are one of the most versatile and commercialized inorganic nanofillers for PP [8,9,10,11,12].

However, on account of high specific surface area and surface energy, CaCO_3_ nanofillers highly tend to agglomerate in a polypropylene matrix and form stress-concentration points, which can dramatically deteriorate the mechanical performance of the formed nanocomposites [11,13]. In order to improve the dispersion of CaCO_3_ nanofillers in PP, organic compounds (such as surfactants, coupling agents, and polymers) are used to modify the surface of CaCO_3_ nanofillers [14,15,16,17]. As a result, the surface energy and polarity, as well as the surface chemical composition of CaCO_3_ nanofillers, can be adjusted to be similar to those of a PP matrix to improve their compatibility, wettability, and adhesion, and the enhancement of mechanical performance for PP is achieved accordingly. For instance, Zhao et al. employed dodecyl dihydrogen phosphate (DDP) to modify spherical CaCO_3_ nanofillers, and successfully changed the CaCO_3_ nanofillers from hydrophilic to lipophilic, which obviously improved the dispersion and mechanical properties of prepared CaCO_3_/PP nanocomposites [14]. Chan et al. modified CaCO_3_ nanofillers with various amounts of stearic acid to toughen PP. The results showed that the nanocomposites filled with monolayer-coated CaCO_3_ nanofillers had the best impact strength, owing to the superior dispersion of the nanofillers in the PP matrix [15]. Similarly, Zaman et al. coated CaCO_3_ nanofillers using pimelic acid, which was also beneficial to the dispersion of CaCO_3_ nanofillers in the PP matrix, leading to improved toughness [16].

However, another important property, the mechanical strength (such as tensile or flexural strength), is often in conflict with toughness in PP composites [17]. Up to now, the reported works have almost suggested that, although the addition of various CaCO_3_ nanofillers could contribute to the improvement in impact toughness, it caused the loss of mechanical strength [8,18]. Furthermore, with the existing technological means, it is also difficult to manufacture high-performance polymer nanocomposites with high loading of inorganic particles, which is crucial for the application of structural or functional polymers and the cost reduction of polymer nanocomposites.

In addition, the above modification methods of CaCO_3_ nanofillers are based on organic modifiers that are harmful for the environment and human health, and should be avoided as much as possible. Besides, there are some other modification methods associated to coating polymers on CaCO_3_ nanoparticles, which commonly require sophisticated synthesis containing multi-step reaction procedures, and lead to high production cost and low production efficiency [19,20]. Furthermore, it is still challenging work to toughen polypropylene with high loading of CaCO_3_ nanofillers and without a negative effect on its strength [21]. Therefore, from the perspective of economy, health, and performance, more CaCO_3_/PP nanocomposites with cost-effective manufacturing, environment friendliness, and well-combined toughness and strength urgently need to be developed.

Our previous work has shown that organic modifiers with complexation ability can improve the fluidity of inorganic nanoparticles and their interfacial bonding with a polymer matrix, and are helpful for enhancing the mechanical performance of PP [22,23]. In the present study, CaCO_3_ nanoparticles (CC) were modified by tartaric acid (TA), a kind of food-grade complexing agent, and used to enhance the mechanical performance of PP. As far as we know, this was the first PP nanocomposite reinforced by tartaric acid-modified CaCO_3_ nanofillers (TAMCC). It is also noteworthy that the impact toughness and mechanical strength of the nanocomposite were superior to those of neat PP even with the high addition TAMCC (50 wt.%). Based on the characteristics of micromorphology, melting behavior, and crystal structure, the toughening and strengthening mechanisms of the nanocomposite were studied. This study can provide an effective way and theoretical support for developing low-cost, safe, and high-performance polypropylene nanocomposites.

## 2. Materials and Methods

**Figure 1:**
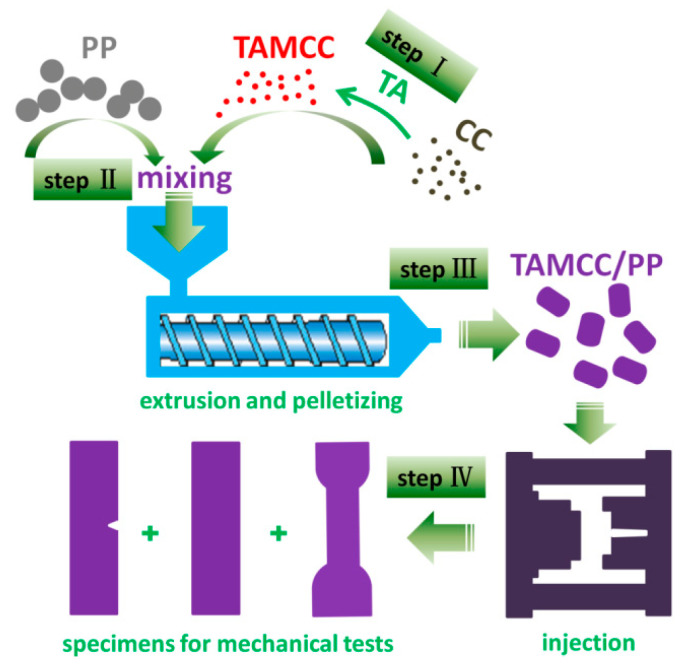
Schematic diagram of the overall research approach.

Herein, in an effort to simultaneously strengthen and toughen polypropylene (PP) by use of CaCO_3_ nanofillers, a green approach was employed. The overall research approach can be described briefly as below (Figure 1). Step I, CaCO_3_ nanofillers (CC) were modified by tartaric acid (TA), a kind of food-grade complexing agent, under certain reaction conditions. Step II, the TA-modified CC (TAMCC) was uniformly mixed with PP pellets. Step III, the mixture of PP and TAMCC was processed into granular TAMCC/PP nanocomposite by means of the extrusion and pelletizing process via a twin-screw extruder. Step IV, the specimens for mechanical tests were manufactured by injection process. Besides, the measurement of mechanical performance, physical and chemical characterizations were also performed to carry out studies of structure, properties, and relevant mechanisms.

### 2.1. Materials

The polypropylene (PP) pellets with a trade mark of PPH-T03 were supplied by Jingmen Branch of China Petrochemical Corporation (Jingmen, China). The CaCO_3_ nanofillers (CC) (with density of 2.7 g cm^–3^ and average diameter of 25 nm) were supplied by Foshan Shunde Yufeng Powder Material Co. Ltd. (Foshan, China). The tartaric acid (TA), namely, 2,3-dihydroxysuccinic acid, was purchased from Sinopharm Chemical Reagent Co. Ltd. (Shanghai, China). All chemicals were of commercial grade and used without further treatments.

### 2.2. Modification of CaCO_3_ Nanofillers

The CC (99.5 g) was dried at 80 °C for 4 h, and then mixed with 100 mL of absolute ethanol in a glass flask to form slurry. TA (0.5 g) was dissolved into 10 mL of absolute ethanol in another container, followed by adjusting pH value to 7 with dilute NaOH solution. After the prepared slurry and TA solution were heated to 40 °C, the TA solution was dropped into the slurry under stirring for 1 h. Subsequently, the mixture was poured out and dried, and the products (tartaric acid-modified CaCO_3_ nanofillers, TAMCC) were obtained after the grinding treatment.

### 2.3. Preparation of CaCO_3_/PP Nanocomposites

The TAMCC and PP were blended through the extrusion and pelletizing process via a twin-screw extruder (L/D ratio = 32, screw diameter of 21.7 mm) (SHJ-20, Nanjing Giant Machinery Co., Ltd., Nanjing, China) under seven barrel temperatures of 200/205/210/215/225/220/200 °C, and then the granular CaCO_3_/PP nanocomposites were prepared. In the experiment, various samples with different weight percentages of TAMCC in nanocomposites were prepared. In order to make comparison, the nanocomposites of unmodified CaCO_3_ nanofillers and PP were also prepared. The samples of nanocomposites of TAMCC and PP, nanocomposites of unmodified CaCO_3_ nanofillers and PP, and neat PP were represented as TAMCC/PP, CC/PP, and PP, respectively.

### 2.4. Measurement of Mechanical Performance

Dumbbell-shaped and rectangular bars were prepared as specimens for tensile and flexural tests using the injection process on a vertical injection molding machine TY-400 (TAYU Machinery Co., Ltd., Hangzhou, China). The tensile tests (GB1040) were performed on a TCS-2000 Universal Testing Machine at room temperature at a crosshead speed of 50 mm/min. In the flexural tests (GB9341), a three-point loading system was used, and the support span length was adjusted to 60 mm. The crosshead speed was 2 mm/min. As for the notched Izod impact experiments, a single-edge 45° V-shaped notch (tip radius of 0.25 mm and depth of 2 mm) was milled in the rectangular bars (the same as rectangular bars for the flexural test). The impact strength measurement was performed according to GB1843. All of the reported values were the average values of five individual measurements.

### 2.5. Characterization

The measurement of Fourier transform infrared spectroscopy (FTIR) was performed on a Nicolet 6700 spectrometer (Thermo Fisher, Waltham, MA, USA) using the KBr pellet technique in the range between 4000 and 500 cm^–1^. The melting behavior was studied by differential scanning calorimetry (DSC) on an STA 409 PC/4/H Luxx apparatus (NETZSCH, Selb, Germany) under a nitrogen atmosphere at a heating rate of 20 °C/min. The XRD spectra were recorded on an X-ray diffractometer (D/MAX-RB, Rigaku, Tokyo, Japan) employing Cu-Ka radiation (λ = 0.15418 nm). The observation of micromorphology of fracture surfaces of the Izod specimen was performed on the Field emission scanning electron microscope (FE-SEM) (JSM-5900LV, JEOL, Tokyo, Japan) with an accelerating voltage of 10 KV. The neat PP, CC/PP, and TAMCC/PP were hot melted and cast on the glass slide to form films, respectively. The surface morphological features of the films were measured using an atomic force microscope (AFM, NX-10, Park Systems Corp., Suwon, Korea). The three-dimensional topographic images and average surface roughness (Ra) of different samples were collected at a scan rate of 1.0 Hz for a scan size of 10 × 10 μm^2^ using the accompanying software (XEI, ver 1.8.2. Build1, Park Systems Corp., Suwon, Korea). In addition, phase images were also collected to evaluate the dispersions of inorganic fillers in nanocomposites. The CC and TAMCC were pressed into tablets under high pressure of 20 MPa for 5 min, and the contact angles of water droplets on the samples were measured on a contact angle meter (JC200D). The melt flow rate (MFR) was measured on an MFR tester (SRZ-400D, Changchun Intelligent Instrument Equipment Co., Ltd., Changchun, China) under a load of 2.16 kg at 210 °C.

## 3. Results

**Figure 2:**
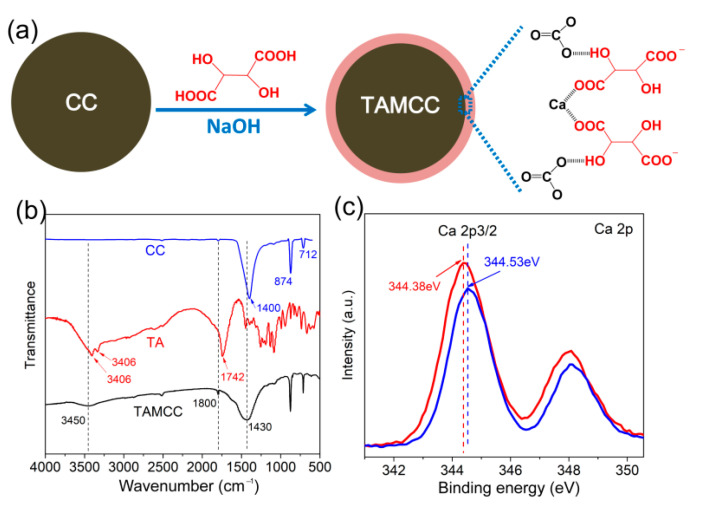
(**a**) Schematic diagram of modification of CC by TA. (**b**) FTIR spectra of CaCO_3_ nanofillers, TA, and TMACC. (**c**) XPS spectra (Ca 2p 3/2 doublet) of CC and TMACC.

As described above, it is an effective approach to improve mechanical performance by means of CC modified with organic compounds. However, for the modification of CC, complicated synthetic processes or harmful ingredients are always needed. In the present study, the TA (a kind of food additive) with low molecular weight of 150.09 g·mol^–1^, and two carboxyl and hydroxyl groups per molecule, was employed, and the CC was modified via the facile process as shown in Figure 2a. In brief, the TA was firstly neutralized using sodium hydroxide (NaOH), and then the neutralized TA solution was mixed with CC. By means of the strong interaction between TA and CC, for instance, hydrogen bonding between –OH (TA) and C–O (CC), complexation between –COO^−^ (TA) and Ca (CC), the modification was facilely fulfilled.

Figure 2b shows the FTIR spectra of CC, TA, and TAMCC. In the characteristic peaks of CC, the bands located at 1400, 874 and 712 cm^–1^ are attributable to the stretching, and out-of-plane and in-plane bending vibrations of C–O, respectively [24]. The characteristic peaks of TAMCC were nearly similar with those of CC, whereas two new bands at 3450 and 1800 cm^–1^ appeared. These two peaks should be ascribed to the modification of TA, corresponding to stretching vibrations of –OH and C=O, respectively [25]. It is noteworthy that compared with neat TA and CC, the bands at 3450, 1800 and 1430 cm^–1^ underwent blue shifts in TAMCC, which should have been due to the formation of hydrogen bonding between C–O and –OH, and complexation between carboxyl groups and Ca, as shown schematically in Figure 2a. Moreover, the chemical interaction on the surface of CaCO_3_ nanofillers can be further demonstrated via XPS spectrum (Figure 2c). It can be seen that the bonding energy of the Ca 2p3/2 doublet for TMACC (344.53 eV) were higher than that of neat CC (344.38 eV). The higher bonding energy in TMAC also corresponded to the increased oxidation state of Ca caused by the complexation between carboxyl groups and Ca, further demonstrating the successful modification of CC by TA [26,27].

**Figure 3:**
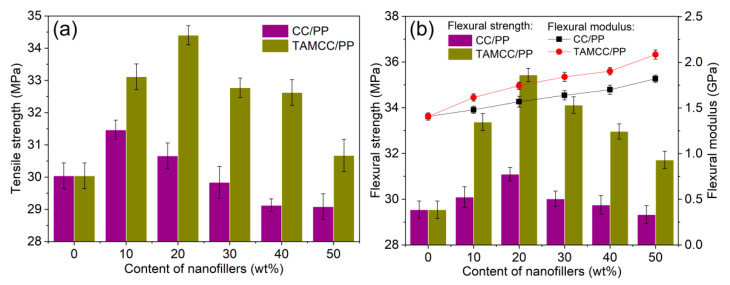
The tensile (**a**) and flexural (**b**) properties of CC/PP and TAMCC/PP with various contents of nanofillers.

Figure 3 shows the evaluation of the tensile and flexural properties of CC/PP and TAMCC/PP. The tensile strength of neat PP was nearly 30 MPa, and it could be improved by the addition of CC or TAMCC (Figure 3a). The reason behind this should have been that the nano-sized CC could provide a large surface area to contact PP. For the CC/PP, it exhibited the highest tensile strength with CC content of 10 wt.%. Nevertheless, the tensile strength underwent considerable degradation if the CC content was higher than 10 wt.%, and the tensile strength of CC/PP was completely lower than neat PP when the CC content was higher than 30 wt.%. It may have been caused by the agglomeration of CC, which could have brought stress concentrators in the PP matrix. For the TAMCC/PP, it showed fairly higher tensile strength than neat PP and CC/PP. For instance, the TAMCC/PP achieved the highest tensile strength of 34.4 MPa, superior to neat PP (30.0 MPa) and CC/PP (30.7 MPa). Additionally, when the TAMCC content was as high as 50 wt.%, the tensile strength of TAMCC/PP was still superior to that of neat PP. The similar result was also obtained in flexural strength evaluation (Figure 3b), further revealing the advantage of TAMCC. In the case of flexural modulus, it could be seen that both CC and TAMCC could play positive roles in the flexural modulus of nanocomposites, which rose with the increment of the content of CC or TAMCC. It is noted that when the contents of nanofillers (CC and TAMCC) were higher than 20 wt.%, with increasing nanofiller contents, the tensile and flexural strengths declined, whereas the flexural modulus kept upgrading (Figure 3b). This may have been on account of two reasons. Above all, the nanofillers with high content would tend to agglomerate and form stress concentrators, and the tensile and flexural strengths of nanocomposites were sensitive to the stress concentrators so as to reduce. Besides, the flexural modulus was evaluated in the elastic deformation zone under a relative low load, which was bearable for the adhesion at the interface between nanofillers and PP even where the nanofillers were agglomerated. This is also consistent with previous reported results. Zhang et al. found that Young’s modulus of polylactide (PLA) significantly increased with the addition of organically modified montmorillonite (MMT), while the tensile strength increased initially and then decreased if the addition of MMT was higher than 5 wt.% [28]. Ghasemi et al. found that the addition of CaCO_3_ nanofillers into the blend of polypropylene and maleic anhydride-grafted polypropylene (PP/MAPP) could improve tensile modulus by 26%, however, the tensile strength declined by 13% [21].

**Figure 4:**
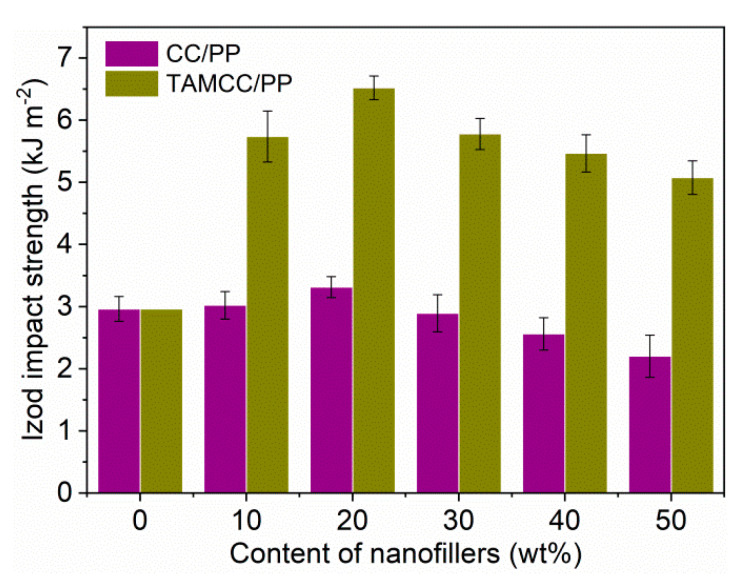
The impact strength of CC/PP and TAMCC/PP with various contents of nanofillers.

The impact strength of CC/PP and TAMCC/PP versus the content of nanofillers is plotted in Figure 4, which displays that both impact strengths of CC/PP and TAMCC/PP were higher than that of neat PP, revealing the toughening effect of nanofillers, i.e., CC and TAMCC. This improved toughness should be principally attributed to the interfacial debonding between nanofillers and the PP matrix, as the same as the toughening mechanism of rubber using rigid nanofillers. In brief, the CC and TAMCC initially acted as stress concentrators, and then they were subject to stress at the triaxial state. Subsequently, the debonding arose at the interface between nanofillers and the PP matrix, followed by the formation of voids surrounding the nanofillers. In the end, the voids facilitated the release of stress and yielding of PP molecular chains, and shear yielding was achieved and led to huge adsorption of energy upon fracture [29]. Afterwards, Chan et al. utilized the high-molecular-weight PP to manifest another toughening mechanism, showing that strong ligaments with high fracture stresses were beneficial to the stabilization of the crack-initiation process and the following energy dissipation at crack-initiation stage, resulting in high impact strength [30]. More importantly, it could be also found that the impact strength of TAMCC/PP was greatly superior to that of CC/PP. For instance, comparing with neat PP, the TAMCC/PP improved 120% in impact strength with an addition of 20 wt.% TAMCC, whereas the CC/PP increased only 12% with the same content of CC. Furthermore, with increasing content of TAMCC up to 50 wt.%, the TAMCC/PP still retained high impact strength (76% higher than neat PP). However, the impact strength of CC/PP with 50 wt.% CC dropped to a value lower than that of neat PP. To the best of our understanding, this prominent toughening of TAMCC should be explained by its good dispersion in the PP matrix.

**Figure 5:**
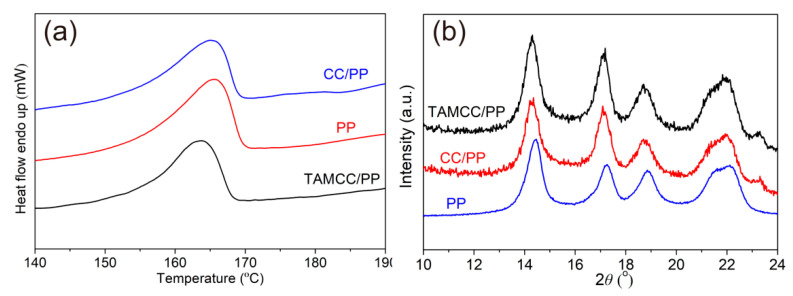
(**a**) DSC melting curves of neat PP, CC/PP, and TAMCC/PP. (**b**) XRD patterns of neat PP, CC/PP, and TAMCC/PP.

In addition to interfacial debonding between nanofillers and PP, the degree of crystallinity and the crystal form of PP also play crucial roles in the toughness of PP nanocomposites. Thumsorn et al. intensively studied the relationship between crystallization features and mechanical properties of cockleshell-derived CaCO_3_ (CS)-filled PP. The results showed that the CS could distinctly promote the formation of β-form PP, leading to improvement in rigidity and toughness of nanocomposites. In their comparative experiment, stearic acid-treated CS would not generate the formation of β-form PP, but could significantly affect the nucleation process and therefore hindered crystallization [31]. Figure 5a shows the DSC melting curves of neat PP, CC/PP, and TAMCC/PP (both CC/PP and TAMCC/PP contain 20 wt.% nanofillers), from which the melting enthalpy (∆*H*_m_) and melting temperature (*T*_m_) could be collected. Furthermore, the degree of crystallinity (*X*_c_) can be calculated by Equation (1) [32]:

*X*_c_ = 100% × ∆*H*_m_/(*W*∆*H*_o_) (1)

where *W* is the weight fraction of PP in nanocomposites, 80 wt.%, and ∆*H*_o_ is the melting enthalpy of a perfect PP crystal, 207 J/g [33,34]. The data of ∆*H*_m_, *T*_m_, and calculated *X*_c_ are shown in Table 1. It was found that all of ∆*H*_m_, *T*_m_, and *X*_c_ decreased in the order of PP, CC/PP, and TAMCC/PP. This should be attributed to the blocking effect of nanofillers on the PP molecular chain, which destroyed the regularity of the PP molecular chain. In our opinion, the TAMCC possessed better dispersion in PP, thus resulting in more PP molecular chains being hindered by this blocking effect, so the decrease in ∆*H*_m_, *T*_m_, and *X*_c_ of TAMCC/PP was most obvious. This was also the reason contributing to higher impact toughness in TAMCC/PP.

Furthermore, it could be also found that all of the DSC melting curves of neat PP, CC/PP, and TAMCC/PP manifested only a single melting peak at the temperature higher than 160 °C, which was the representative melting peak of α-PP, suggesting that the addition of CC or TAMCC could not result in the formation of any other crystal types in PP [31,35,36]. As is well known, PP can be categorized into three classes based on the crystal form, i.e., the monoclinic, trigonal, and orthorhombic types, corresponding to α-, β-, and γ-PP, respectively. Due to the superior toughness and heat distortion temperature, β-PP has been identified as the ideal choice for manufacturing high-performance PP nanocomposites. Mai et al. prepared β-PP nanocomposites using nano-CaCO_3_ with β-nucleation (β-CC) as nanofillers. It could be found that a new melting peak at ~150 °C belonging to β-PP obviously arose on the DSC melting curve if the β-CC was introduced. Owing to the synergistic toughening effect of nano-CaCO_3_ and β-PP, the resultant β-PP nanocomposites achieved high impact strength and stiffness [37]. However, in the present study, the DSC melting curves of CC/PP and TAMCC/PP did not show the melting peak of β-PP, indicating that CC and TAMCC could not toughen PP by the formation of β-PP. In order to confirm that β-PP was not formed in CC/PP and TAMCC/PP, the XRD patterns of neat PP, CC/PP, and TAMCC/PP were measured (Figure 5b). There were five peaks at 2θ = 14.5°, 17.2°, 18.8°, 21.2°, and 22.1° in the XRD pattern of neat PP, corresponding to (110), (040), (130), (111), and (131) planes of α-PP, respectively [8,36,38]. It could be found that both XRD patterns of CC/PP and TAMCC/PP were similar with that of neat PP without showing the representative peaks of β-PP, further verifying that both CC and TAMCC could not toughen PP by the formation of β-PP as described above.

The melt flow rate (MFR) is a crucial parameter for the processibility of polymeric materials. Normally, for the same materials, the higher MFR value corresponds to the better mobility and processibility. Table 2 shows the measured MFR for neat PP, CC/PP, and TAMCC/PP (both CC/PP and TAMCC/PP contain 20 wt.% nanofillers). It could be seen that the TAMCC/PP exhibited the highest MFR value among the three samples, implying its superior mobility and processibility. It was also probably owing to the improved dispersion of TAMCC in the PP matrix, which was beneficial to blocking the intermolecular contact and friction of PP chains. In other words, TAMCC with good dispersion acted as the lubricant in PP, leading to improved mobility and MFR of TAMCC/PP. In turn, better mobility would also promote the dispersion of nanofillers, i.e., TAMCC.

**Figure 6:**
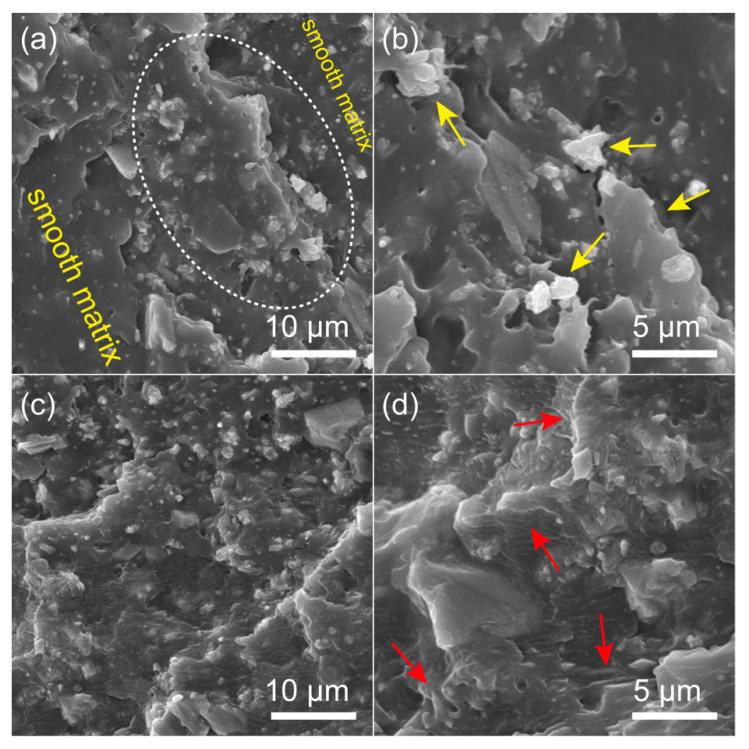
SEM images of impact fracture surfaces for CC/PP (**a**,**b**) and TAMCC/PP (**c**,**d**).

To manifest the dispersion of nanofillers and fracture features of the prepared PP nanocomposites, the micromorphologies of the impact fracture surface of CC/PP and TAMCC/PP (both samples contained 20 wt.% nanofillers) were observed using FE-SEM. Figure 6a shows that the impact fracture surface of CC/PP principally consisted of two regions, the smooth matrix region and collection region of agglomerates (indicated by the dotted ellipse), suggesting the poor dispersion of CC in the PP matrix. In the enlarged SEM image (Figure 6b), it could be found that the fracture surface exhibited lots of large agglomerates, which had inferior compatibility with the matrix and introduced a large amount of cavity flaws and stress concentration points in the nanocomposite, as indicated by the yellow arrows. By contrast, the impact fracture surface of TAMCC/PP displayed a continuous and undulating morphology without uneven distribution (Figure 6c), revealing the improved dispersion of TAMCC in PP. Figure 6d shows that matrix ligaments (indicated by the red arrows) were formed, indicating that the matrix underwent plastic deformation, and the energy generated by shear yield could be absorbed during impact fracture, so that the toughness of TAMCC/PP was improved. Moreover, it was hard to find the agglomerated nanoparticles on the fracture surface, further demonstrating the improved dispersion and interface adhesion of TAMCC in PP. From the above observation and comparison, it can be concluded that TA was an effective modifier to improve the dispersion and interface adhesion of CC in the PP matrix, which was also confirmed in our related experiments [22,23], so that the negative effect of stress concentration was tremendously eliminated. As a result, a great improvement in both strength and toughness was achieved for TAMCC/PP.

**Figure 7:**
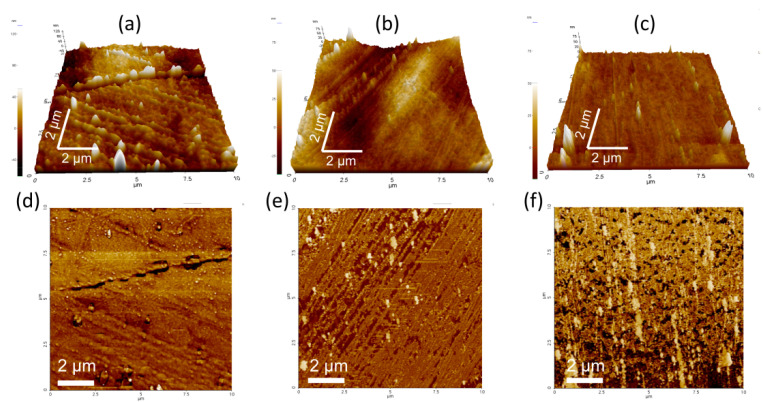
Three-dimensional topographic images (**a**–**c**) and phase images (**d**–**f**) of neat PP (**a**,**d**), CC/PP (**b**,**e**), and TAMCC/PP (**c**,**f**).

Figure 7 shows the AFM images of neat PP, CC/PP, and TAMCC/PP. The three-dimensional topographic images (Figure 7a–c) reveal that the neat PP exhibited the roughest surface (with the highest Ra values of 12.1 nm). With addition of CC (20 wt.%), the surface of CC/PP became smoother (Ra = 11.7 nm), because the nano-sized CC could fill the gap between PP aggregates to form a smoother surface. The TAMCC/PP with 20 wt.% TAMCC manifested the smoothest surface and the lowest Ra values (4.2 nm) among the three samples, which should be attributed to the improved fluidity and dispersion of TAMCC in the PP matrix. Meanwhile, the neat PP appeared uniform brown in the phase image (Figure 7d). In the case of CC/PP and TAMCC/PP, there was not only the brown matrix, but there were also aggregated long strips (Figure 7e) and scattered dots (Figure 7f), respectively. Apparently, the scattered dots further demonstrate the improved dispersion of TAMCC comparing with CC (aggregated long strips). Thus, the results of AFM were well consistent with the observation of SEM.

**Figure 8:**
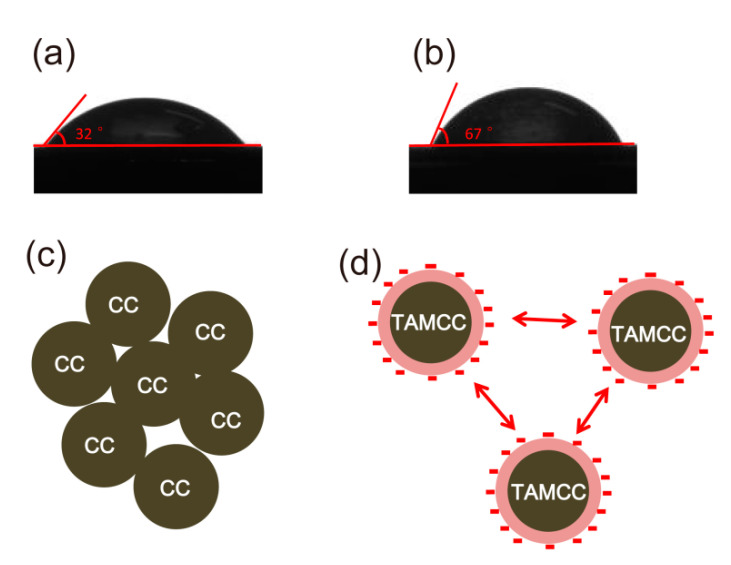
Comparison of the contact angles of water droplets on TAMCC (**a**) and CC (**b**); schematic diagram of dispersion of CC (**c**) and TAMCC (**d**) in PP.

It is well known that the most commonly used organic modifier, e.g., stearic acid, could lower surface polarity and improve their compatibility and dispersion in PP. In the present study, the organic modification was also identified as an important contribution of the improved compatibility and dispersion of TAMCC in PP. The surface polarities of different samples were characterized using contact angles of water droplets on their surfaces. Figure 8a,b show that the contact angles of water droplets on the TAMCC and CC were 32° and 67°, respectively, revealing the better hydrophobicity, viz., lower polarity, of TAMCC, which was greatly beneficial to the improvement in the compatibility and dispersion of TAMCC in the nonpolar PP matrix.

It should also be noted that the TA containing two carboxyl and hydroxyl groups per molecule belonged to strong polar organic compounds, and the TA modification could only provide limited hydrophobicity for TAMCC. To the best of our knowledge, the superior dispersion of TAMCC should also have originated from some other contributions besides the improved compatibility. In the synthesis of TAMCC, the TA was firstly neutralized using NaOH, and then the TAMCC was formed via a strong interaction, i.e., hydrogen bonding and complexation, between TA and CC. Actually speaking, the CC particles were coated by sodium tartrate instead of tartaric acid. Thus, the TAMCC exhibited a negative outer surface, which would allow TAMCC with improved dispersion in the PP melt under the repulsive interaction in between negative nanofillers. However, in the case of CC, this repulsive interaction was absent, and the nanofillers highly tended to agglomerate due to high specific surface area and surface energy. The corresponding schematic diagram is shown in Figure 8c,d.

To quantitatively illustrate the influence of nanofillers’ dispersion on mechanical performance of PP nanocomposites, the relation between tensile strength and interfacial interaction is characterized using Equation (2) [39]:

(2) $$\sigma_{y}=\sigma_{\mathrm{ym}}\frac{1-\varphi_{f}}{1+2.5\varphi_{f}}\;\exp\;(B_{\sigma y}\varphi_{f})$$

where *σ*_y_ and *σ*_ym_ are yield stresses of the nanocomposite and polymer matrix; *φ*_f_ is the volume fraction of inorganic filler, and; *B*_σy_ is the parameter representing the interfacial interaction between inorganic filler and the polymer matrix. Ordinarily, the better interfacial interaction will lead to a higher *B*_σy_ value. Herein, the samples of CC/PP and TAMCC/PP both containing 20 wt.% nanofillers are taken for example. Assuming that CC and TAMCC possessed the same density, the volume fractions (*φ*_f_) of CC and TAMCC in nanocomposites were the same. The yield stress of the polymer matrix (*σ*_ym_) of CC/PP was also equal to that of TAMCC/PP. Thus, the difference of yield stresses (*σ*_y_) for CC/PP and TAMCC/PP was just caused by the *B*_σy_ value. As measured above, the tensile strength of TAMCC/PP (34.4 MPa) was superior to that of CC/PP (30.7 MPa), when the content of nanofiller was 20 wt.%. Given that, under the same testing conditions, higher tensile strength corresponded to the larger yield stress, the TAMCC/PP possessed better interfacial interaction than CC/PP obtained through Equation (2). To the best of our understanding, the enhanced interfacial interaction in TAMCC/PP is attributable to improved dispersion and compatibility as described above, which was beneficial for forming more interaction points and stronger interaction forces between TAMCC and the PP matrix. However, for CC/PP, due to poor dispersion and compatibility, there were lots of agglomerates and cavity flaws (Figure 6), which could have impeded the interfacial interaction between CC and the PP matrix, resulting in the unsatisfactory mechanical performance.

## 4. Conclusions

In summary, nano-CaCO_3_ was modified using a green modifier (TA) and used as nanofillers to synchronously strengthen and toughen polypropylene. The successful modification was confirmed using FTIR and XPS spectra. The tensile, flexural, and impact strengths were completely evaluated, and the results showed that enhanced strength and toughness were simultaneously achieved in the obtained nanocomposites (TAMCC/PP). For instance, when the content of nanofillers was 20 wt.%, the TAMCC/PP not only exhibited obviously higher tensile strength (34.3 MPa) than neat PP (30.0 MPa), but also improved impact strength by 120%. Furthermore, the mechanical strength and impact toughness of TAMCC/PP with ultra-high content (50 wt.%) of nanofillers were still superior to those of neat PP. The measurements of DSC and XRD suggested that the toughening mechanism was attributed to interfacial debonding and decreased crystallinity, but without the formation of β-PP. The measurement of MFR revealed that the MFR value of TAMCC/PP (2.38) was higher than those of CC/PP (2.12) and neat PP (2.18), implying its superior mobility and processibility among the three samples. Meanwhile, the observations of FE-SEM and AFM demonstrated the compatibility and dispersion of TAMCC in the PP matrix, which was considerably helpful for achieving high mechanical performance in TAMCC/PP nanocomposites.

Overall, enhanced mechanical strength and impact toughness were simultaneously achieved in the present TAMCC/PP nanocomposites even with high content (50 wt.%) of nanofillers. Meanwhile, the high content of low-cost CaCO_3_ nanofillers was beneficial for cost reduction of PP nanocomposites, and food-grade modifier (TA) can help the TAMCC/PP to be applied into the fields of food, medical and health industries, etc. Therefore, owing to well-balanced strength and toughness, low-cost, and safety, the TAMCC/PP shows great application potential in the field of high-performance polymer nanocomposites.

## Figures and Tables

**Table 1:** Comparison of *T*_m_, ∆*H*_m_, and *X*_c_ for neat PP, CC/PP, and TAMCC/PP.

Sample	*T_m_* (°C)	∆*H*_m_ (J/g)	*X*_c_ (%)
Neat PP	165.632	71.656	34.285
CC/PP	164.971	63.587	24.339
TAMCC/PP	163.638	59.983	22.96

**Table 2:** Comparison of MFR for neat PP, CC/PP, and TAMCC/PP.

Sample	Neat PP	CC/PP	TAMCC/PP
MFR (g∙10 min^−1^)	2.18	2.12	2.38

## Data Availability

The data presented in this study are available on request from the corresponding author.
